# Role of Bacterioferritin & Ferritin in *M. tuberculosis* Pathogenesis and Drug Resistance: A Future Perspective by Interactomic Approach

**DOI:** 10.3389/fcimb.2017.00240

**Published:** 2017-06-08

**Authors:** Divakar Sharma, Deepa Bisht

**Affiliations:** Department of Biochemistry, National JALMA Institute for Leprosy and Other Mycobacterial DiseasesAgra, India

**Keywords:** bacterioferritin, ferritin, protein-protein interaction, aminoglycosides resistance, *M. tuberculosis*

## Abstract

Tuberculosis is caused by *Mycobacterium tuberculosis*, one of the most successful and deadliest human pathogen. Aminoglycosides resistance leads to emergence of extremely drug resistant strains of *M. tuberculosis*. Iron is crucial for the biological functions of the cells. Iron assimilation, storage and their utilization is not only involved in pathogenesis but also in emergence of drug resistance strains. We previously reported that iron storing proteins (bacterioferritin and ferritin) were found to be overexpressed in aminoglycosides resistant isolates. In this study we performed the STRING analysis of bacterioferritin & ferritin proteins and predicted their interactive partners [ferrochelatase (hemH), Rv1877 (hypothetical protein/probable conserved integral membrane protein), uroporphyrinogen decarboxylase (hemE) trigger factor (tig), transcriptional regulatory protein (MT3948), hypothetical protein (MT1928), glnA3 (glutamine synthetase), molecular chaperone GroEL (groEL1 & hsp65), and hypothetical protein (MT3947)]. We suggested that interactive partners of bacterioferritin and ferritin are directly or indirectly involved in *M. tuberculosis* growth, homeostasis, iron assimilation, virulence, resistance, and stresses.

## Introduction

*Mycobacterium tuberculosis* is the causing factor of tuberculosis (TB) etiology and remains one of the top 10 causes of death worldwide in 2015. Recently WHO reported 10.4 million new TB cases and 1.8 million deaths worldwide (WHO Report, 2016). Due to disappointment of BCG vaccine in adults, chemotherapy through potent anti-TB drugs is the last option to reduce prevalence and mortality but unfortunately, emergence of drug resistant tuberculosis such as multi drug resistant tuberculosis (MDR-TB), extensively drug resistant tuberculosis (XDR-TB) and totally drug resistant tuberculosis (TDR-TB) have made chemotherapy complicated. Aminoglycosides are the second line drugs of choice used especially for the treatment of MDR-TB along with the fluoroquinolones. Aminoglycosides and fluoroquinolones are the only option for the treatment of MDR-TB (with some side effects as well as less efficacy as compared to first line drugs). When the usual treatments are not possible than recently approved drugs (bedaquiline and delamanid) have been used for the treatment of MDR TB, XDR-TB and TDR-TB (with more side effects as compared to first and second line drugs). In *M. tuberculosis* they typically inhibit protein synthesis by interacting with protein translational machinery. Two dimensional gel electrophoresis coupled with mass spectrometry is the best accepted approach for expression proteomics (Lata et al., 2015a,b; Sharma et al., 2015b, 2016b; Sharma and Bisht, 2016). Since last decade a panel of proteomics and bioinformatics studies related to aminoglycosides resistance have been accumulated (Sharma et al., 2010, 2014, 2015b,a, 2016a,b; Kumar et al., 2013; Sharma and Bisht, 2017a,b). Here we emphasized on the *M. tuberculosis* iron storage proteins (bacterioferritin and ferritin) and their interactive protein partners which might be involved in pathogenesis, virulence and drug resistance.

Iron is an essential entity for metabolism of the biological cells and hence crucial for the chemistry of life. Iron assimilation, storage and their utilization play a crucial role not only in pathogenesis/pathobiology (growth, survival, virulence and latency) but also in emergence of aminoglycosides drug resistance strains of *M. tuberculosis* (Reddy et al., 2012; Kumar et al., 2013; Sharma et al., 2015b). Recently, Khare et al. (2017) reported that iron storage proteins are involved in maintaining iron homeostasis in *M. tuberculosis* (Khare et al., 2017). Bacterioferritin (Rv1876) and ferritin (Rv3841) are unique to maintain iron storage as well as homeostasis in *M. tuberculosis*. In mycobacteria, various genes products and their interactive partners required for high affinity iron acquisition have been identified such as siderophore production, uptake of ferric-siderophores, production of iron storage proteins and uptake of heme. Production, storage and function of iron uptake mechanisms are controlled by a regulatory protein IdeR (Gold et al., 2001). Heme is the preferable iron source for *M. tuberculosis* and its acquisition is done by a biosynthetic enzyme ferrochelatase (Rv1485). Rv1485 catalyzes the last step of heme biosynthesis in which iron is interleaved to protoporfhyrin IX to form protoheame (Dailey and Dailey, 2002). It is essential because it supplies the heme which is a preferred iron source for *M. tuberculosis* (Parish et al., 2005) and serves as a cofactor for various metabolic enzymes such as catalase-peroxidase and DosS/DosT two component system [active sites contains heme] (Svistunenko, 2005).

In pathogenic mycobacteria, usually high affinity systems are essential for maintenance of an infection, virulence and resistance. Greater definition of the functions of both the identified genes and their products, ferritin (*bfr*B) and bacterioferritin (*bfr*A) will refine our understanding of mycobacterial iron acquisition and the interplay between components of the iron systems have increased intensities under iron-rich and decreased intensities under iron deprived conditions (Pandey and Rodriguez, 2012). Under iron-rich conditions, *M. tuberculosis* represses iron acquisition and induces iron storage proteins suggesting the significant role of iron storage proteins in iron homeostasis. *M. tuberculosis* usually synthesizes two iron storage proteins: ferritin (*bfr*B) and bacterioferritin (*bfr*A). *Bfr*B is mandatory to overcome iron limitation and defense against oxidative stress, whereas *bfr*A is superfluous for victorious adaptation to those stresses. Studies of *M. tuberculosis* lacking *bfr*B gene (which encode the ferritin an iron storage protein) reported increased intracellular concentration of iron. They depicted that increasing iron concentration (absence of bacterioferritin and ferritin) decreased resistance to various anti-TB drugs including aminoglycosides as well as fluoroquinolones (Pandey and Rodriguez, 2012) and suggested that ferritin and bacterioferritin are not only mandatory to maintain iron homeostasis but also make *M. tuberculosis* resistant to aminoglycosides. Kurthkoti et al suggested that iron-dependent regulator IdeR induces ferritin (bfrB) expression by alleviating Lsr2 repression in *M. tuberculosis* (Kurthkoti et al., 2015). *Whi*B7 (Rv3197A) is Fe-S cluster-bound protein (transcriptional regulatory protein), which not only associates with aminoglycosides resistance but also predicted to be in the IdeR|Rv2711 regulon (Gold et al., 2001; Morris et al., 2005). Recently Kuberl et al. (2016) suggested that pupylation machinery maintains the iron homeostasis by targeting iron storage proteins (Kuberl et al., 2016).

In our previous studies of expression proteomics we have discovered, that bacterioferritin (Rv1876) and ferritin (Rv3841) were overexpressed in aminoglycosides (amikacin and kanamycin) resistant *M. tuberculosis* clinical isolates (Kumar et al., 2013; Sharma et al., 2015b). Molecular docking revealed that aminoglycosides drugs (AK and KM) bind to conserved bacterioferritin domain of Rv1876 and ferritin domain of Rv3841 and suggested that overexpression of these proteins might be to neutralize/modulate the drug effect and could be involved in aminoglycosides resistance mechanisms of *M. tuberculosis* (Kumar et al., 2013; Sharma et al., 2015b). Although the enzymes and their connected pathways involved in iron metabolism in *M. tuberculosis* are well recognized, still our information related to iron transportation, trafficking, iron dependent post-transcriptional & translational regulations and protein-protein interactions in mycobacteria is inadequate. Recently Sharma et al. (2016a) reported that inducible overexpression of recombinant ferritin in *E. coli* (BL21) leads to shift in MIC of AK & KM and suggested their probable roles in conferring aminoglycosides resistance (Sharma et al., 2016a). Consequently, proteins involved in iron storage, assimilation, regulation, uptake and their utilization can be a promising anti-mycobacterial targets against the drug resistant tuberculosis.

## Bacterioferritin and ferritin protein-protein interaction: unlock the secrets of iron related pathways in aminoglycosides drug resistance

STRING-10 is an online server which is used to predict the interacting partners of the iron storage proteins [bacterioferritin (Rv1876) and ferritin (Rv3841)] (Sharma et al., 2016a,b; Sharma and Bisht, 2017a). “STRING uses a combination of prediction approaches and an integration of other information (neighborhood, transferred neighborhood, gene fusion, co-occurrence, co-expression, experiments, databases, text mining). Network was made at medium confidence level (0.400) allowing all active prediction methods, which corresponds to approximately 50% possibility of association” (Sharma et al., 2016a,b). In the network display, each node represents a protein, and each edge represents an interaction.

**Figure 1:**
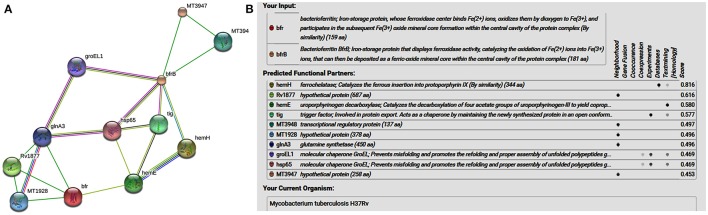
**(A)** STRING analysis reveals the interaction partners of *M. tuberculosis* H37Rv bacterioferritin and ferritin protein. **(B)** The score for each interaction partner is assigned and shown in the table inbuilt in figure. The highest score for hemH association was found to be 0.816 followed by Rv1877, a probable transporter (its confidence score of association was 0.616). The confidence score for association for tig corresponds to 0.577, and for molecular cheprone (groEL1 & hsp65) was found to be 0.469.

STRING analysis predicted (Figure 1) that ferrochelatase (hemH), Rv1877 (hypothetical protein/probable conserved integral membrane protein), uroporphyrinogen decarboxylase (hemE) trigger factor (tig), transcriptional regulatory protein (MT3948), hypothetical protein (MT1928), glnA3 (glutamine synthetase), molecular chaperone GroEL (groEL1 & hsp65), and hypothetical protein (MT3947) were functional partners of the bacterioferritin and ferritin at medium confidence level (0.400) allowing all active prediction methods. Interactome analysis suggested that molecular chaperone GroEL (*gro*EL1 & *hsp*65) and trigger factor (tig) were co-expressed as well as experimentally reported. *In silico* analysis predicted trigger factor (tig) protein as a novel target for interaction with ferritin protein. Trigger factor (tig) is not only involved in eliciting the expression of proteins & their export but also helps in maintaining the open conformation of newly synthesized protein (chaperone activity).

## Prediction of pupylation sites

Using the default threshold (medium) with cutoff 2.452; GPS-PUP predicted pupylation sites in bacterioferritin and ferritin proteins which were tabulated in Table 1.

**Table 1:** Predicted / identified pupylation sites in bacterioferritin and ferritin proteins.

**ORF no**.	**Position of lysine residue undergoes pupylation**	**Peptides**	**Score**	**Cut-off**
Rv1876	122	TSAVLLE**K**IVADEEE	2.819	2.452
Rv3841	10	EYEGPKT**K**FHALMQE	2.732	2.452

*K-Lysine undergoes pupylation*.

## Discussion

Emergence of extensively-drug resistant tuberculosis (XDR-TB) is the consequence of interrupted treatment of multi-drug resistant tuberculosis (MDR-TB) with second line anti-tubercular drugs (aminoglycosides and fluoroquinolones). Proteins engage in iron storage, assimilation, regulation, uptake and their utilization could not only be involved in *M. tuberculosis* pathobiology, growth, virulence and latency but also in aminoglycosides drug resistance and might be potential anti-mycobacterial drug target against the drug resistant tuberculosis (Reddy et al., 2012; Kumar et al., 2013; Sharma et al., 2015b, 2016a; Khare et al., 2017). Pandey and Rodriguez also suggested that ferritin is not only mandatory to maintain iron homeostasis in *M. tuberculosis* but also ferritin deficient bacilli are more susceptible to killing by antibiotics (Pandey and Rodriguez, 2012). Our previous studies reported that bacterioferritin (Rv1876) and ferritin (Rv3841) were overexpressed in aminoglycosides (amikacin and kanamycin) resistant *M. tuberculosis* clinical isolates and suggested their involvement in resistance (Kumar et al., 2013; Sharma et al., 2015b). Recently Sharma et al. (2016a) reported that inducible over expression of recombinant ferritin in *E. coli* (BL21) increased the MIC shift of AK & KM and make the bacteria more resistant against the aminoglycosides drugs (Sharma et al., 2016a). These findings suggested its probable roles in conferring resistance.

Interactome analysis of bacterioferritin (Rv1876) and ferritin (Rv3841) by STRING-10 also suggested that bacterioferritin and ferritin protein interacted with their partners such as ferrochelatase, hypothetical protein (Rv1877), uroporphyrinogen decarboxylase, trigger factor, transcriptional regulatory protein (MT3948), hypothetical protein (MT1928), glutamine synthetase, Molecular chaperones (groEL1 & Hsp65), and hypothetical protein MT3947 which were involved in intermediary metabolism and respiration, cell wall and cell processes, virulence, detoxification, adaptation, conserved hypotheticals and regulatory proteins. Hypothetical protein (Rv1877), a Probable conserved integral membrane protein, ferrochelatase and trigger factor might be potential novel targets predicted by bacterioferritin and ferritin protein-protein interaction. Rv1877 having 14-transmembrane helixes (TMH), possibly involved in transport of drug across the membrane and could be a potential drug target against the aminoglycosides drug resistance. Rv1877 is the homologous of *lfr*A and deletion of this gene increased the susceptibility against various antibiotics including the aminoglycosides in mycobacteria (Li et al., 2004). Recently, Mehra et al. (2016) reported that Rv1877 is the part of ABC transporter and predicted its involvement in drug resistance (Mehra et al., 2016). Ferrochelatase involved in heme biosynthetic pathways is a preferred iron source for *M. tuberculosis*. Trigger factor which not only having chaperone activity but also involved in eliciting the protein expression and their export which could maintain the 3D conformation of newly synthesized proteins and prevents misfolding as well as promotes the refolding of unfolded polypeptides generated under stressed conditions. Bhuwan et al. (2016) reported and validated the STRING in the interaction of RipA with Chaperone MoxR1 (Bhuwan et al., 2016). Sharma et al. (2015b) reported that trigger factor, bacterioferritin and ferritin were overexpressed in aminoglycosides resistant *M. tuberculosis* clinical isolates (Sharma et al., 2015b) and suggested that trigger factor might trigger expression of bacterioferritin and ferritin and their interactive partners which could be involved in aminoglycosides drug resistance. We suggested that cumulative effect of iron storage proteins (bacterioferritin and ferritin) and their interactive partners {hypothetical protein (Rv1877), ferrochelatase, trigger factor and others} might be involved in various stress, and aminoglycosides drug resistance. Predicted pupylation sites in bacterioferritin and ferritin also suggested its involvement not only in iron homeostasis but also in aminoglycosides resistance (Kuberl et al., 2016; Sharma et al., 2016a). Further detailed and in-depth investigations of these interactome and pupylome could explore the aminoglycosides resistance and might be used as potential drug targets against this issue.

## Author contributions

DS design the concept and wrote the manuscript. DS and DB finalized the manuscript.

### Conflict of interest statement

The authors declare that the research was conducted in the absence of any commercial or financial relationships that could be construed as a potential conflict of interest.
